# Biochar influences on soil CO_2_ and CH_4_ fluxes in response to wetting and drying cycles for a forest soil

**DOI:** 10.1038/s41598-017-07224-6

**Published:** 2017-07-28

**Authors:** Mark S. Johnson, Cameron Webster, Rachhpal S. Jassal, Iain Hawthorne, T. Andrew Black

**Affiliations:** 1 Institute for Resources, Environment and Sustainability, University of British Columbia, 418-2202 Main Mall, Vancouver, British Columbia V6T 1Z4 Canada; 2 Department of Earth, Ocean and Atmospheric Sciences, University of British Columbia, Vancouver, BC Canada; 3 Faculty of Land and Food Systems, University of British Columbia, Vancouver, BC Canada

## Abstract

Biochar has been the focus of significant research efforts in agriculture, but little research has been conducted in forested ecosystems. Here, we assess CO_2_ and CH_4_ fluxes from a forest soil in response to biochar additions using a before-after-control-intervention experimental design. Soil CO_2_ and CH_4_ fluxes were measured over a series of wetting cycles by coupling soil mesocosms equipped with auto-chambers to a laser-based spectrometer for high-frequency measurements of gas fluxes and related soil processes. We found that soil CO_2_ fluxes were higher and CH_4_ fluxes were less negative (e.g. reduced CH_4_ uptake) for the biochar-amended soil compared to the no biochar condition. Furthermore, biochar improved soil infiltrability under wet conditions, and enhanced soil moisture levels under dry conditions. Biochar additions shifted the point of maximum soil respiration (i.e. soil CO_2_ efflux) to a slightly wetter soil moisture level. The point of maximum CH_4_ uptake was also shifted to a slightly wetter moisture level for soil with biochar. Overall differences in soil gas fluxes were found to be minor compared to the increase in soil carbon resulting from the biochar addition. Biochar may thus contribute to improved forest management through increases to soil carbon stocks and improved soil moisture levels.

## Introduction

Almost 200,000 ha of forest are harvested and replanted in British Columbia each year, which has a significant impact on the soil carbon (C) stocks of managed forest landscapes. Management of soil C is thus crucial for improving carbon sequestration, reducing net emissions of greenhouse gases, and improving the sustainability of managed forest landscapes. Sustainable forest management is predicated upon sustainable management of forest soil, with soil C content a key factor in forest soil health^1^. Given the increasing importance of production forests for providing woody materials and non-timber forest products for supply chains rather than primary (old-growth) forests^2^, maintaining or enhancing soil C in managed forest ecosystems is crucial for the long term viability of these systems. Forests in coastal British Columbia that are actively managed primarily for timber production without consideration of soil C stocks have experienced soil C depletions of more than one-third during the transition from old-growth to a mature, managed forest stand^3^.

Although carbon sequestration is a key priority for British Columbia, where a forest carbon offset protocol is already in place^4^, burning forest harvest slash material to facilitate replanting and reduce fire risk remains a common practice for managed forest landscapes in the province, releasing ~8 Mt CO_2_ yr^−1^ into the atmosphere^5^. Converting harvest slash into biochar for application to forest soils during stand re-establishment could help with seedling growth^6^, forest restoration^7^, and reductions in the environmental impacts of forest management^8^.

As a soil amendment derived from waste biomass sources via pyrolysis, biochar has garnered significant attention as a potential strategy to improve agricultural soil management^9^. Biochar use in agricultural soils has been shown to increase crop yields in many (but not all) systems^10^, and to improve the water holding capacity of agricultural soils^11^. Biochar also represents a global negative emission potential of 0.7 Pg C yr^−1^ ^12^, and has been shown to be effective for reducing soil fluxes of greenhouse gases in some contexts^13^. Research has shown that biochar additions can contribute to the stabilization of root-derived organic C, increasing the stable C content in soil^14^. Some studies have found that biochar additions can stimulate the decomposition of soil organic matter (SOM) in a process known as the “priming effect”^15^. However, a recent meta-analysis found that biochar additions more commonly depresses SOM mineralization, generally resulting in a negative priming effect, except in sandy soils^16^.

Biochar use may provide opportunities to improve the sustainability of forest soil management. We designed a controlled experiment to evaluate the impact of biochar additions to a forest soil from a managed forest in coastal British Columbia on soil gas fluxes of carbon dioxide (CO_2_) and methane (CH_4_). The study employed a state-of-the-art cavity ring-down spectrometer (CRDS) to simultaneously measure CO_2_ and CH_4_ fluxes from soil mesocosms in relation to a series of wetting cycles under controlled laboratory conditions. Previous research has demonstrated hysteresis on a diurnal basis between soil CO_2_ fluxes and soil temperature in forested ecosystems, assuming that soil moisture varies at longer time scales^17^. Here, we varied soil moisture at a sub-daily time scale while keeping soil temperature constant, and conducted synchronous measurements of soil CO_2_ and CH_4_ fluxes in order to evaluate relationships between soil gas fluxes and soil moisture levels in response to biochar additions. We hypothesized that biochar additions would affect soil CO_2_ and CH_4_ fluxes by influencing soil moisture content.

## Materials and Methods

### Experimental Design

We designed a laboratory-based before-after-control-intervention (BACI) experiment^18^ to test the influence of biochar additions on soil fluxes of CO_2_ and CH_4_ in response to wetting and drying cycles. The soil for the experiment was collected from a Douglas-fir (*Pseudotsuga menziesii* (Mirbel) Franco var. *menziesii*) forest located near the east coast of central Vancouver Island near Campbell River, British Columbia (BC), Canada (49.87°N, 125.33°W). The uppermost litter – fibric – humic (LFH) layer and overlying organic layer (0–10 cm) were removed to produce a more homogeneous soil, and one that reflects soil conditions following logging. Mineral soil was then collected and sieved to less than 10 mm in the field, removing course roots and plant debris. The soil was then transported to the laboratory where it was air dried and sieved to less than 4 mm. The sieved humo-ferric podzol soil had 74.5% sand, 18.5% silt and 7.0% clay, with 3.1% C.

The sieved soil was used to fill two mesocosms that were instrumented with soil sensors and connected to an automated soil gas flux measuring system described below. The mesocosms were placed in the Environmental Interfaces Laboratory, a constant-temperature (20 °C) facility at the University of British Columbia. The soil mesocosms were then put through eight wetting cycles over four months in order to compare soil fluxes of CO_2_ and CH_4_ between the two mesocosms. The fluxes and soil moisture dynamics during this period provided the background relationships between the mesocosms against which the influence of biochar addition could be determined. Following this pre-treatment period, biochar was added to the surface soil of one mesocosm, with soil gas fluxes measured for both mesocosms over a series of eight bi-weekly wetting cycles to simulate winter conditions, followed by two longer drying cycles of three-weeks each to simulate summer conditions.

**Figure 1:**
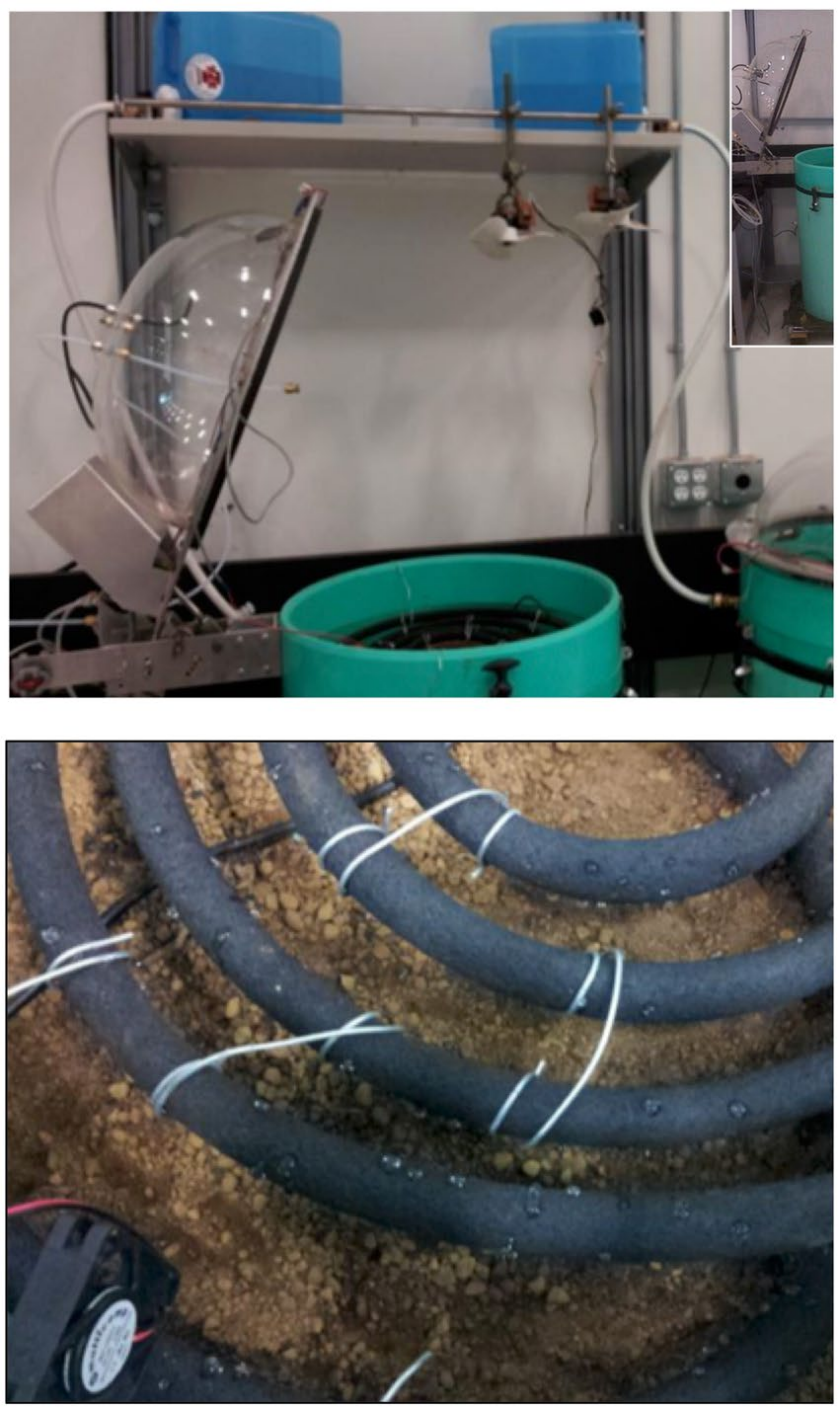
Soil mesocosms with auto-samplers and watering systems (upper panel, with inset photo showing full mesocosms); detail of soaker hose used for rainfall simulation (lower panel). The fan beside the soaker hose ensured air mixing within the chamber headspace during flux measurements.

PVC cylinders (0.52 m internal diameter cut into 0.75 m lengths) were lined at the bottom with a 1 cm metal mesh topped with landscaping fabric, and placed on a wire metal shelf over wood supports (Fig. 1). The cylinders received 10 cm of washed gravel (20 mm crushed rock), topped by 15 cm of screened soil saved from laboratory sieving (>4 mm and <10 mm), and finally 50 cm of sieved <4 mm soil. The coarser material underlying the upper 50 cm provided a lower horizon reflecting site conditions and drainage. The total dry mass of the forest soil was approximately 146 kg per mesocosm. Care was taken when packing the soil to achieve a uniform bulk density of approximately 1.35 g cm^−3^, similar to the field condition for the humo-ferric podzol (bulk density of 1.353 ± 0.51 g cm^−3^ at the 10–80 cm depth, and a volumetric coarse fraction of 31% greater than 2 mm^19^).

Soil volumetric water content (*θ*), soil temperature (*T*_*s*_), and electrical conductivity (EC) were measured (sensor model GS3 (*θ*, *T*_*s*_ and EC), Decagon Devices, Pullman, WA USA) in each mesocosm at the 7.5 cm depth. The sensors were connected to a data logger (CR1000, Campbell Scientific, Logan UT USA) that also controlled the opening and closing of the auto-chambers as described below.

### Soil Flux Measurements in Relation to Biochar Additions

The soil mesocosms were topped with auto-chambers programmed to alternately close for 4 min at half-hour intervals during which time chamber air was circulated through a laser-based CRDS (Model G2301-f, Picarro Inc.) enabling synchronous determinations of CO_2_ and CH_4_ fluxes^20^. Following the mesocosm stabilization and background flux measurement periods, 433 g of biochar, equivalent to an application rate of 20 t ha^−1^, was added to one mesocosm. Biochar was carefully mixed into the upper 5 cm of soil using a small garden rake which avoided disturbing the sensors installed at the 5–10 cm depth. Biochar was produced by Diacarbon Inc. (Burnaby, BC Canada) from 2 cm chipped pieces of Douglas-fir slash feedstock that were pyrolyzed for 30 min at 420 °C. The biochar was sieved to <2 mm; the sieved fraction consisted of 78.8% C on a dry matter basis with low volatiles (18.8%) and ash (2.4%), and a near neutral pH (6.86 ± 0.04) compared to the sieved mineral soil (pH of soil: 5.6 ± 0.05).

### Watering System

A watering system was installed in each of the chambers. These systems consisted of a soaker hose (Gardena, product number 59-7448-6) shaped into a spiral using wire, suspended 5 cm above the soil surface and connected to a water canister with a 10 L capacity (Fig. 1). The soaker hose was connected to the water canister via tubing passing into the mesocosm through a port in the side of the chamber collar. The port was sealed with rubber gaskets to avoid air exchange when the chambers were closed. As water drained from the container through the soaker hose, small water droplets fell onto the soil at an application rate of 2 L hr^−1^, equivalent to a rainfall rate of 9.4 mm hr^−1^ for the 52 cm diameter columns. Each wetting event consisted of 6 L of simulated rainfall (28.3 mm) that was evenly distributed onto the surface of the soil while continual soil flux measurements were made. Prior to the experiment, the soaker hose was flushed with sufficient water such that the concentration of dissolved organic C concentration exiting the hose was statistically similar to water entering the hose.

### Before-After-Control-Intervention (BACI) Experimental Details

The soil mesocosms were identically prepared, which would theoretically allow them to be directly compared to assess treatment effects for the mesocosm receiving biochar relative to the control mesocosm without biochar. However, measured gas fluxes and soil moisture dynamics during the period prior to biochar application were found to differ between the mesocosms (Table 1), confirming the appropriateness of the BACI^18^ approach to analyzing treatment effects. All relationships during the pre-treatment period were highly linear and highly significant (Table 1). Differences in the measured parameters integrated the systemic differences between the soil mesocosm flux measurement systems (e.g., the individual mesocosms and associated watering systems, sensors, auto-chambers and connections to the CRDS). These systemic differences remained constant across pre-treatment and post-treatment periods.

**Table 1:** Relationships between the two mesocosms during pre-treatment period for measured parameters.

Soil CO_2_ flux (μmol CO_2_ m^−2^ s^−1^)	y = 0.81x + 0.13	R^2^ = 0.92, p < 0.001
Soil CH_4_ flux (nmol CH_4_ m^−2^ s^−1^)	y = 0.96x − 0.17	R^2^ = 0.79, p < 0.001
Soil volumetric moisture (Θ, cm^3^ cm^−3^)	y = 1.26x − 6.2	R^2^ = 0.92, p < 0.001

These relationships were used to compute the “No Biochar” condition for the treated mesocosm using the Before-After-Control-Intervention (BACI) experimental design.

In the BACI analysis, the relationships between mesocosms during the background period (including wetting cycles) were used to model the fluxes anticipated for the treatment mesocosm in the absence of the treatment. The difference between measured and anticipated fluxes for the treatment mesocosm thus reflect the effect of biochar additions on the fluxes of CO_2_ and CH_4_. Measured parameters from the mesocosm receiving biochar are referred to as “Biochar” in the Results and Discussion, while values reported as “No Biochar” were computed based on measured parameters from the control mesocosm during the treatment period and the parameter-specific relationships between the mesocosms during the pre-treatment period reported in Table 1. The post-treatment period consisted of more than 4000 individual flux measurements of CO_2_ and CH_4_ for each mesocosm.

In order to compare soil gas fluxes in C terms against the C added via biochar, we subtracted the mean flux for the “No Biochar” treatment from the mean flux for the “Biochar” treatment for CO_2_ and CH_4_. These were then converted to kg C ha^−1^ yr^−1^ based on the molecular weight of C in each mole of CO_2_ and CH_4_. Further, we assessed the climatic influence of biochar additions relative to the soil gas fluxes by summing their fluxes in CO_2_ equivalent (CO_2_e) terms based on the 100-year radiative forcings of CH_4_ and CO_2_, where CH_4_ is 34 times that of CO_2_ in CO_2_e terms^21^.

### Statistical Analyses

Differences between treatments for soil gas fluxes of CO_2_ and CH_4_ were compared parametrically by Student’s t-test after first assessing normality by the Shapiro-Wilk test of normality. Differences were considered significant for p < 0.05. We also compared the soil gas fluxes non-parametrically by plotting the cumulative distributions of the full soil gas flux datasets. All analyses were conduct in R version 3.3.3^22^ with figures generated using ggplot2^23^.

## Results

### Soil moisture

**Figure 2:**
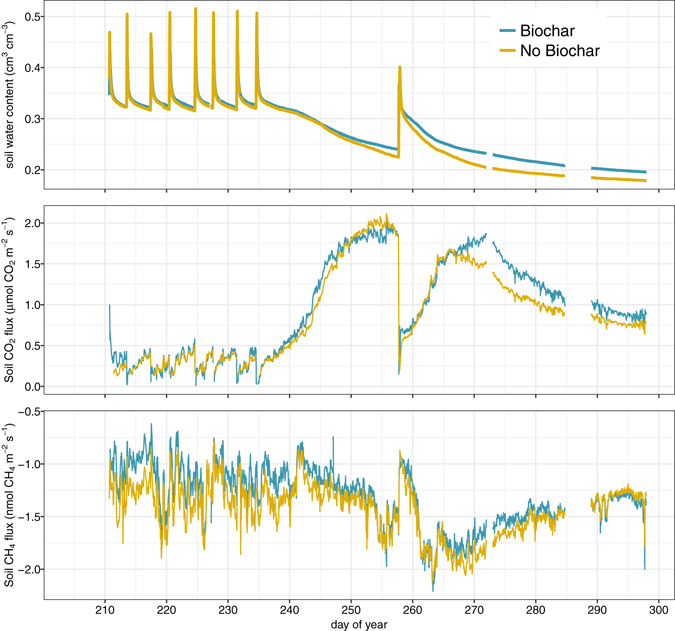
Temporal dynamics of soil water content and soil gas fluxes of CO_2_ and CH_4_ for Biochar and No Biochar conditions.

The experiment was structured to simulate rainfall patterns typical of coastal British Columbia with wetting cycles occurring in rapid succession for the wet season, and drying phases interrupted by occasional wetting for the dry season. Soil moisture levels responded rapidly to the wetting events, although soil with biochar retained additional soil moisture during drying phases (Fig. 2). With biochar, the minimum soil moisture after 40 days of drying was 10% greater compared to soil moisture without biochar. During wetting events, however, maximum soil moisture was 12% lower for soil with biochar at peak wetness. Having lower water content at peak wetness and greater water content under dry conditions suggests that biochar additions to this forest soil improved aeration and infiltrability under wet conditions, and improved water availability under dry conditions.

### CO_2_ fluxes

**Figure 3:**
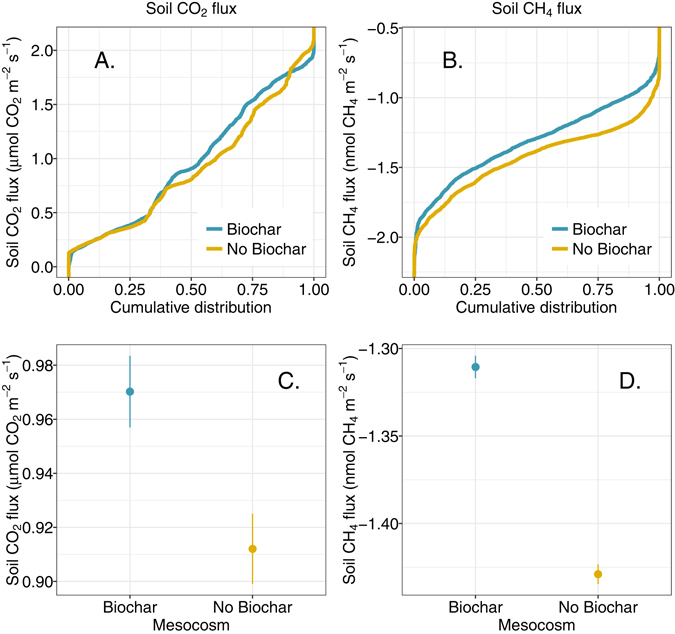
Cumulative distribution of soil CO_2_ effluxes (**A**) and soil CH_4_ fluxes (**B**) for Biochar and No Biochar conditions over the wetting and drying cycles. Mean soil gas fluxes (±1 standard error) are presented for CO_2_ (**C**) and CH_4_ (**D**).

Adding biochar to the forest soil resulted in generally increased soil CO_2_ effluxes (0.97 ± 0.01 μmol CO_2_ m^−2^ s^−1^ for soil with biochar vs. 0.91 ± 0.01 μmol m^−2^ s^−1^ without biochar; values as means ± 1 standard error (SE), p < 0.01, Fig. 3). Biochar additions also shifted the point of maximum soil respiration to slightly wetter soil moisture level. Higher soil CO_2_ effluxes were observed for soil without biochar at soil moisture levels less than the point of maximum soil CO_2_ efflux, but for soil moisture levels above the point of maximum soil CO_2_ efflux, higher effluxes were observed for biochar-amended soil. Maximum soil CO_2_ effluxes were very similar for both conditions (2.0 μmol CO_2_ m^−2^ s^−1^ without biochar and 2.1 μmol CO_2_ m^−2^ s^−1^ with biochar).

**Figure 4:**
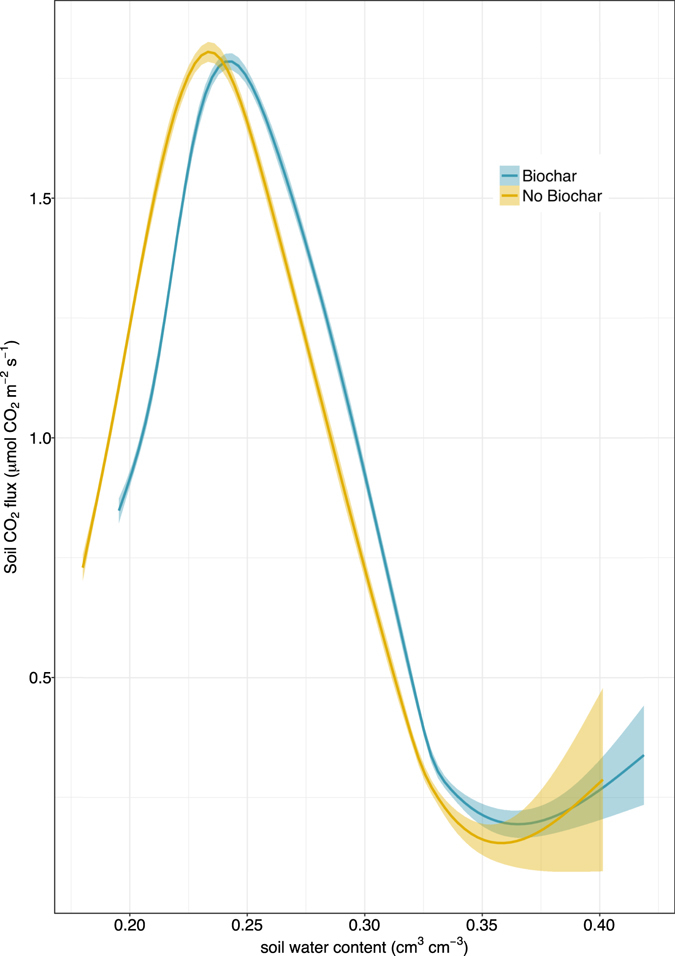
Soil CO_2_ effluxes as a function of soil water content for Biochar and No Biochar conditions.

An initial pulse of CO_2_ was observed for the biochar-amended mesocosm in response to the first wetting event following biochar application (Fig. 2). Following this initial flush of CO_2_, the mesocosms exhibited similar temporal trends, with initial declines in CO_2_ effluxes in response to soil wetting events, followed by increased CO_2_ effluxes as soils dried to the point of maximum soil respiration (Fig. 4), beyond which soil respiration became water limited and CO_2_ effluxes declined. Comparing the distributions of CO_2_ efflux measurements for each mesocosm, CO_2_ effluxes from the biochar-amended soil tended to be higher than for soil without biochar (Fig. 3A).

### CH_4_ fluxes

Soil CH_4_ fluxes were negative throughout the experiment, indicating methane uptake by soil microbes in both mesocosms. Cumulative distributions of the CH_4_ flux measurements for each mesocosm demonstrated that overall CH_4_ fluxes were higher (e.g. less negative) under the biochar-amended condition (Fig. 3B), indicating less methane consumption when biochar was applied to this forest soil (−1.31 ± 0.01 nmol CH_4_ m^−2^ s^−1^ for biochar-amended soil vs. −1.43 ± 0.01 nmol m^−2^ s^−1^ without biochar; mean ± 1 SE, p < 0.001, Fig. 3D).

**Figure 5:**
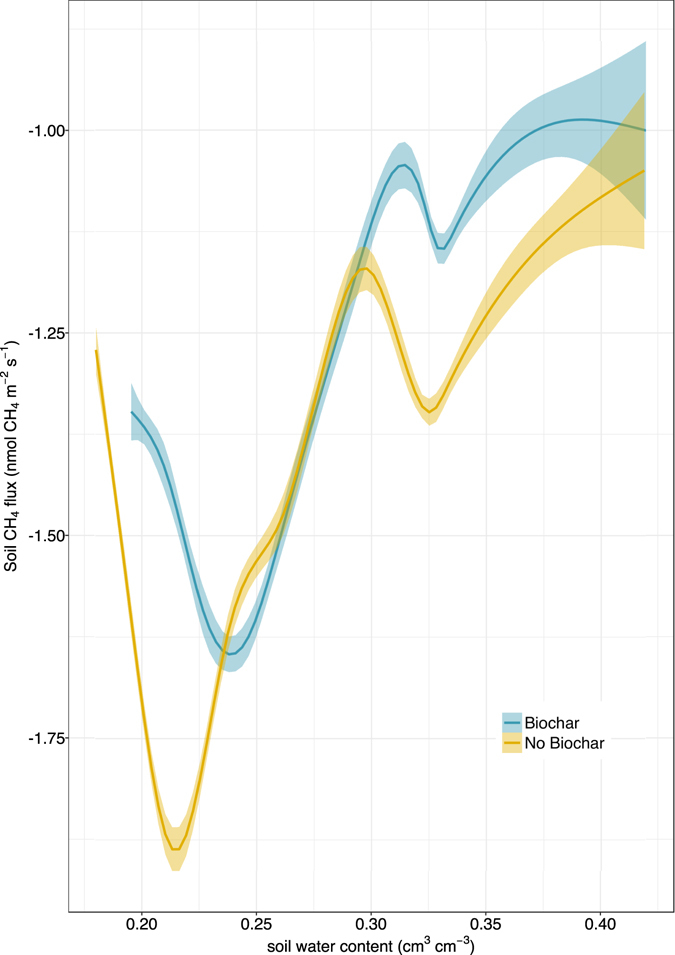
Soil CH_4_ fluxes as a function of soil water content for Biochar and No Biochar conditions.

Both mesocosms exhibited pronounced responses to wetting and drying phases, with maximum CH_4_ uptake occurring at a slightly drier soil moisture level for soil without biochar (Fig. 5), and generally higher CH_4_ consumption (more negative CH_4_ flux values) under the no biochar condition as soils became wetter. In both mesocosms, methane uptake exhibited short-term reductions following wetting (less negative fluxes), followed by increased uptake (more negative values for CH_4_ fluxes) during early stages of drying. As soils dried beyond the point of maximum CH_4_ uptake, fluxes became less negative, indicating reduced CH_4_ uptake under water-limited conditions (Figs 2 and 5).

## Discussion

Biochar use in forest soils has been shown to be useful as a replacement for organic matter and liming agents in relation to forest restoration activities^7^. However, little is known regarding gas fluxes from forest soils in response to biochar additions. These fluxes are of interest as they help to determine the climatic effect of C management in forested ecosystems, where biochar additions can enhance soil C stocks^24^ and improve forest biomass production^7^. Information on soil gas fluxes is still needed in order to determine the full life-cycle impact of biochar additions^13^. Field studies of biochar addition to temperate forest soils are limited, and have not identified significant differences in soil greenhouse gas fluxes between treatments^25^, which could be due to methodological limitations such as the lack of continuous sampling from static chambers. Here, we conducted a controlled laboratory experiment to evaluate the temporal responses of soil gas fluxes to wetting events for a biochar-amended soil, making continuous flux measurements across a wide range of soil moisture levels.

Wetting events quickly induced temporal switches^26^ in soil gas responses, with increasing soil moisture depressing soil CO_2_ effluxes in both biochar and non-biochar conditions. A temporal switch in soil CH_4_ fluxes was only observed in response to the wetting event that followed the longer drying period (Fig. 2). Due to the absence of plants in this experiment, the temporal switches observed were likely more microbial in nature rather than exclusively due to diffusive limitations in gas transfer. This can be surmised from the persistence of the switch effect in gas fluxes relative to the more rapid decline in soil moisture levels (Fig. 2). No statistically significant differences in lag effects between treatments were observed, although the soil CO_2_ pulse in response to the “dry season” wetting event was delayed for the biochar amended soil relative to unamended soil (Fig. 2).

In this experiment, we found the impact of biochar addition on forest soil resulted in an additional 220 kg C ha^−1^ yr^−1^ of soil CO_2_ emissions, plus a reduction in methane uptake rates of 39.4 mol CH_4_ ha^−1^ yr^−1^ (0.47 kg CH_4_-C ha^−1^ yr^−1^). This reduction in methane uptake is equivalent to an emission of 21.5 kg CO_2_e ha^−1^ yr^−1^ at a global warming potential of 34 for methane^21^. These enhanced soil gas fluxes integrate the combined effects of any biochar decomposition with any biochar-related decomposition of native soil organic matter (e.g. the priming effect^16^). Changes in soil redox conditions following biochar additions may be related to some of the changes in soil CH_4_ fluxes, noting that biochar impacts on soil redox reactions can change over time^27^.

We can compare these soil gas fluxes against the biochar added in order to determine the minimum period of climatic benefit that could be expected from the biochar addition. In cases where biochar additions result in negative priming (e.g. reduced SOM decomposition and lower CO_2_ effluxes), this period could be quite long. For our case, in which a positive priming was observed, which is consistent with expectations for a sandy soil^14^, the minimum period of climatic benefit could be less.

Here, the 20 t ha^−1^ biochar with a C content of 78.8% (15.8 t C ha^−1^) expressed in CO_2_e terms would be equivalent to an addition of 57.8 t CO_2_e ha^−1^. In the most conservative comparison in which the soil gas fluxes observed during the three-month experiment were assumed to continue unchanged into the future, the enhanced soil respiration of CO_2_ (807.6 kg CO_2_e ha^−1^ yr^−1^) and the reduced methane consumption following biochar addition to this forest soil (21.5 kg CO_2_e ha^−1^ yr^−1^) together would equal the climatic benefit of the biochar addition (57.8 t CO_2_e ha^−1^) in ~70 years. This minimum period of climatic benefit is similar to other shorter-duration incubation studies^28^. However, the approach to this calculation represents the most conservative estimate from a climate system perspective, noting that the soil gas flux results of this study would likely decline as decomposition of readily available C compounds are rapidly exhausted, yielding a much lower biochar decomposition rate for increasing time after application^16^. Further, our study did not consider the enhanced biomass growth commonly observed in response to biochar additions to forest soils^7^. In particular, it has been shown that biochar can stabilize root-derived carbon^14^, and even when biochar causes a short-term positive priming of native SOM, the biochar remains stable in soils over centuries^16^ to millennial time scales^29^ with a net positive impact on soil C stocks^16^.

Since 2010, British Columbia has harvested an average of 184,000 ha yr^−1^, making it the province with the largest annual forest harvest area in Canada^30^ and nearly 8 Mt CO_2_ emitted from burning of the harvest slash to facilitate transplanting. Biochar may serve as a useful complement to site preparation techniques following forest harvest^31^. Converting even a portion of the 15.5 Mt yr^−1^ of British Columbia clearcut harvest residuals^32^ into biochar for soil application could supply the addition of about 20 t biochar ha^−1^ to replanted areas, which would help to maintain soil C levels that otherwise tend to decline over time in managed temperate forests^3^. Emergent international agreements regarding reporting of environmental impacts related to trade in forest products, including wood pellet exports from British Columbia to Europe and Asia, should account for reductions in soil C stocks over time that result from business-as-usual practices. These frameworks should also acknowledge strategies to improve the sustainability of soil C as a component of forest management, and thereby the viability of managed forest landscapes.

## Data Availability

The datasets generated and analyzed during the current study are available in a Github repository at https://github.com/UBCecohydro/data.public.
